# 1-Piperidine Propionic Acid Inhibits PAR2/SerpinB3 Signaling and Reduces Glioblastoma Tumor Aggressiveness

**DOI:** 10.3390/ijms27167240

**Published:** 2026-08-13

**Authors:** Mariagrazia Ruvoletto, Santina Quarta, Elena Rampazzo, Lorena Lucatello, Roberto Luisetto, Gianmarco Villano, Veronica Di Paolo, Alessandra Biasiolo, Marco Di Pascoli, Luigi Quintieri, Francesca Capolongo, Luca Persano, Patrizia Pontisso

**Affiliations:** 1 Department of Medicine, University of Padova, Via Giustiniani, 2, 35128 Padova, Italy; mariagrazia.ruvoletto@unipd.it (M.R.); santina.quarta@unipd.it (S.Q.); alessandra.biasiolo@unipd.it (A.B.); marco.dipascoli@unipd.it (M.D.P.); patrizia@unipd.it (P.P.); 2 Department of Women’s and Children’s Health, University of Padova, Via Giustiniani, 3, 35128 Padova, Italy; elena.rampazzo@unipd.it; 3 Pediatric Research Institute–IRP, Corso Stati Uniti 4, 35127 Padova, Italy; 4 Department of Comparative Biomedicine and Food Science, University of Padova, Viale dell’Università 16, 35020 Legnaro, Italy; lorena.lucatello@unipd.it (L.L.); veronica.dipaolo@unipd.it (V.D.P.); francesca.capolongo@unipd.it (F.C.); 5 Department of Surgical, Oncological and Gastroenterological Sciences, University of Padova, Via Giustiniani 2, 35128 Padova, Italy; roberto.luisetto@unipd.it (R.L.);; 6 Laboratory of Drug Metabolism, Department of Pharmaceutical and Pharmacological Sciences, University of Padova, Largo Egidio Meneghetti 2, 35131 Padova, Italy; luigi.quintieri@unipd.it

**Keywords:** glioblastoma, 1-piperidine propionic acid, protease-activated receptor-2, SerpinB3, blood–brain barrier

## Abstract

Glioblastoma multiforme is the most aggressive primary brain tumor in adults, which displays extremely poor prognosis. Protease-activated receptor 2 (PAR2) and its downstream effector SerpinB3 are overexpressed in aggressive glioblastomas. In this study we evaluated the antitumor activity of 1-piperidine propionic acid (1-PPA), an allosteric PAR2 inhibitor, in in vitro preclinical models of glioblastoma. PAR2 and SerpinB3 were analyzed at the transcriptional and protein level in glioblastoma cell lines and primary cultures. These were treated with 1-PPA alone or in association with temozolomide (TMZ) and the effects evaluated by Incucyte^®^ technology. Pharmacokinetics and tissue distribution of 1-PPA were assessed in mice by LC-MS/MS. 1-PPA significantly reduced glioma cell proliferation, migration, and invasion, thus promoting apoptotic cell death, in a concentration-dependent manner. The combined treatment with TMZ led to a concentration-dependent decrease in cell proliferation (12–20%) compared to TMZ alone. Molecularly, 1-PPA downregulated *PAR2* and *SerpinB3* expression. Pharmacokinetic studies in healthy mice showed that 1-PPA is systemically bioavailable and distributes to several organs, including the brain. These data indicate that 1-PPA shows brain exposure and capability to affect different hallmarks of aggressiveness in glioblastoma cells, including hyperproliferation and invasion, supporting its further development as a novel therapeutic strategy in these tumors.

## 1. Introduction

Glioblastoma multiforme (glioblastoma) is the most aggressive primary brain tumor in adults, characterized by high cellular heterogeneity, diffuse infiltrative growth, and resistance to conventional therapies [1,2,3]. The current standard of care, consisting of maximal safe surgical resection followed by radiotherapy and concomitant and adjuvant temozolomide (TMZ), provides only limited clinical benefit, with a median overall survival of approximately 12–15 months and five-year survival rates below 10% [2,4,5]. Poor therapeutic outcomes are driven by diffuse infiltrative growth, pronounced intratumoral heterogeneity, intrinsic and acquired therapy resistance [6,7,8], and the restrictive nature of the blood–brain barrier (BBB), which severely limits the delivery of systemically administered agents to the tumor site [9,10]. These limitations highlight the urgent need for novel therapeutic approaches capable of targeting both tumor-intrinsic and microenvironment-dependent mechanisms of glioblastoma aggressiveness.

Increasing evidence indicates that inflammation-driven signaling and tumor–microenvironment interactions play central roles in glioblastoma growth, invasion, immune evasion, and resistance to therapy [11]. Within this context, protease-dependent signaling networks have emerged as key drivers of malignant progression, linking extracellular proteolytic activity and matrix remodeling to intracellular oncogenic pathways [12]. Protease-activated receptor 2 (PAR2), a G protein-coupled receptor activated by extracellular serine proteases, acts as a molecular sensor of the proteolytic microenvironment and promotes tumor cell proliferation, migration, invasion, and survival [13,14,15]. By integrating inflammatory and protease-mediated cues with downstream signaling pathways, such as MAPK/ERK [13,15,16,17], PAR2 contributes to the establishment of a pro-tumorigenic niche and represents an attractive, yet still underexplored, therapeutic target in glioblastoma.

SerpinB3, a member of the serine protease inhibitor family, has been shown to be overexpressed in aggressive glioblastoma and other malignancies, where it supports cancer stem cell maintenance, cellular survival, and resistance to therapy [18,19,20]. Experimental evidence indicates that SerpinB3 depletion enhances apoptotic responses and sensitizes glioblastoma cells to radiotherapy, underscoring its functional relevance in tumor progression and therapy resistance [20]. It is interesting to note that recent findings have identified a positive loop between PAR2 and SerpinB3, since the anti-protease activity of this serpin is essential for PAR2 activation, which in turn induces the synthesis of SerpinB3 by increasing the CCAAT enhancer-binding protein beta (C/EBP-β) transcription factor, known to upregulate the synthesis of SerpinB3 [15,21].

In this context, 1-piperidine propionic acid (1-PPA) has recently emerged as a highly promising small-molecule candidate for targeting PAR2-dependent signaling [22,23]. 1-PPA is an allosteric inhibitor of PAR2 that interferes with receptor activation and downstream signaling cascades, leading to inhibition of SerpinB3 expression [21,22]. Interestingly, 1-PPA has been recently reported to prevent lipid accumulation and liver cancer development in vivo by inhibiting the PAR2/SerpinB3 axis [24]. In addition, it has been demonstrated that pharmacological blockade of PAR2 attenuates neuroinflammation [25] and that its inhibition by 1-PPA reduces neuroglia inflammation and amyloid aggregate formation [23].

In the present study, we investigated the anticancer effects of 1-PPA, including effects on cell proliferation, migration, invasion, and apoptosis in established human glioblastoma cell lines with different expressions of SerpinB3. Furthermore, the pharmacokinetic (PK) profile and tissue distribution of 1-PPA were investigated, with a focus on its oral absorption and brain exposure.

## 2. Results

### 2.1. PAR2 Expression in Glioblastoma Correlates with Tumor Aggressiveness

PAR2 has recently emerged as a key factor in the tumor microenvironment (TME) of glioblastoma, driving tumor progression, angiogenesis, and survival [14,17].

**Figure 1:**
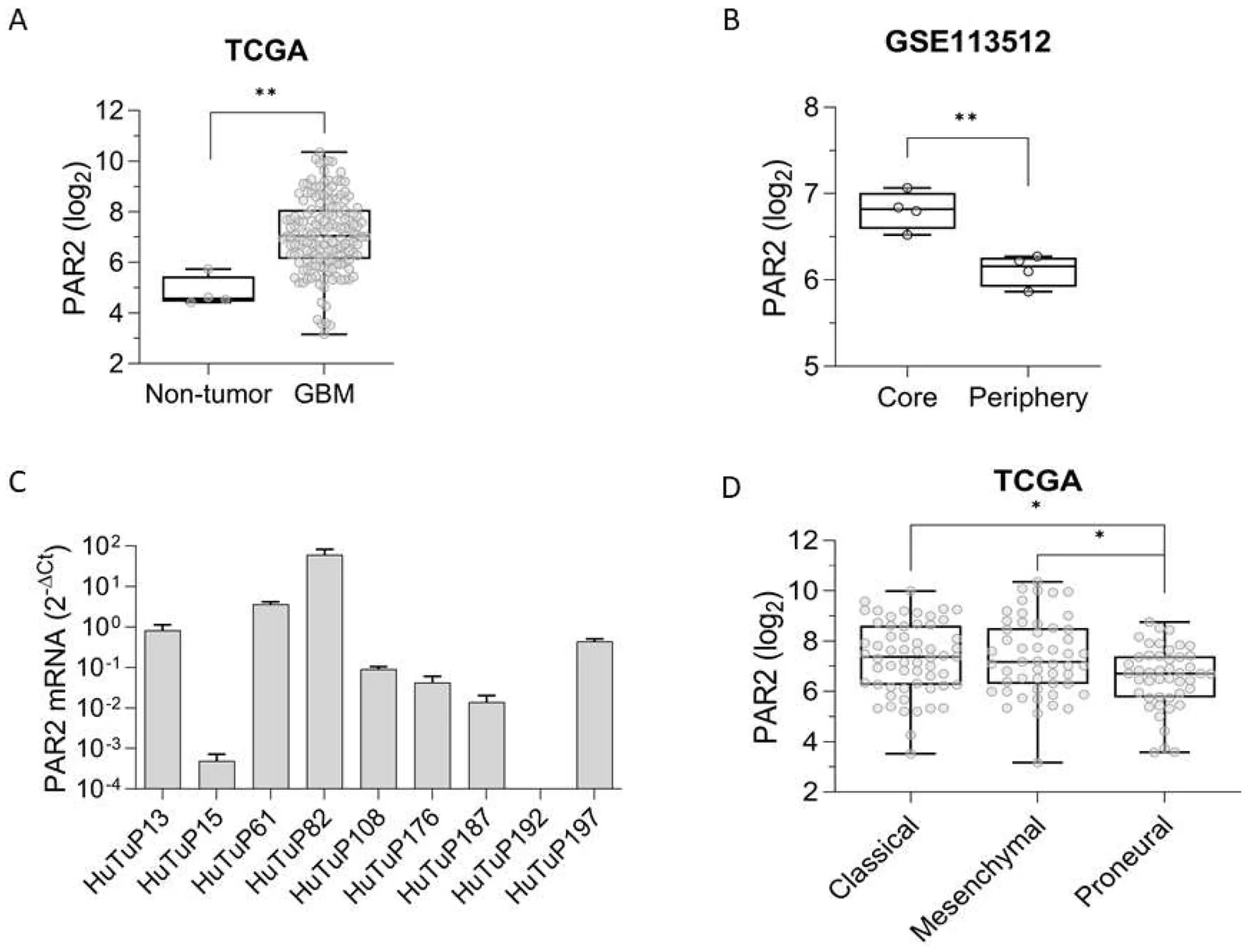
PAR2 expression in glioblastoma. (**A**) Boxplot displaying the mRNA expression of PAR2 in glioblastoma tumors (n = 156) and non-tumor (n = 4) specimens from the TCGA dataset. (**B**) PAR2 expression in samples from glioblastoma core or matched tumor periphery from the GSE113512 dataset (n = 8). (**C**) Bar plot summarizing the expression levels of PAR2 mRNA in nine different glioblastoma primary cultures. (**D**) Boxplot displaying the mRNA expression of PAR2 in glioblastomas from the TCGA dataset, subgrouped according to their molecular subtype. ** *p* < 0.01 by unpaired (Welch correction) or paired Student’s *t*-test in A and B, respectively. * *p* < 0.05 by one-way ANOVA with Tukey’s multiple comparisons test in (**D**).

To corroborate these findings, we performed comprehensive analysis of multiple transcriptomic datasets. Analysis of the TCGA [26] and Murat (GSE7696; [27]) glioblastoma patient cohorts revealed a significant heterogenic upregulation of *PAR2* mRNA in glioblastoma samples (Figure 1A and Figure S1C), a finding further validated by RT-qPCR in glioblastoma primary cell cultures (Figure 1C).

To better investigate the relationship between PAR2 and tumor aggressiveness, we analyzed the GSE113512 dataset [28], which focuses on intratumoral heterogeneity. *PAR2* expression was significantly enriched within the necrotic and hypoxic tumor core (Figure 1B), a niche known to harbor chemoresistant glioblastoma stem cells (GSCs), compared to the more vascularized peripheral areas [29]. Furthermore, comparative analysis using the Gravendeel (GSE16011; [30]), Rembrandt (GSE68848; [31]), and Murat (GSE7696; [27]) datasets demonstrated that *PAR2* levels were consistently higher in glioblastomas than in lower-grade gliomas or non-neoplastic brain tissue (Figure S1A).

According to transcriptomic analyses, glioblastoma tumors can be broadly categorized into proneural, classical, and mesenchymal molecular subtypes, with the mesenchymal phenotype exhibiting the highest invasive potential, inflammatory signaling, and therapeutic resistance [32,33,34]. Intriguingly, we also proved, in several datasets, a significantly reduced expression of *PAR2* in the generally less aggressive proneural tumors (Figure 1D and Figure S1B), although without associations with *IDH* mutations (Figure S1C) [35]. In addition, survival analyses further supported the suggested association between *PAR2* overexpression and tumor aggressiveness, since glioblastoma patients expressing higher levels of *PAR2* correlate with a poorer outcome; however, without reaching statistical significance (Figure S1D) [31,36]. Collectively, these data indicate that *PAR2* expression is associated with high-grade glioblastomas and a hypoxic tumor microenvironment, here potentially favoring their aggressiveness [37,38], as already suggested in rat glioma models [39].

### 2.2. Activation of the SerpinB3/PAR2/ERK1/2 Circuit in Glioblastoma Cells

To investigate the functional interplay between SerpinB3 and PAR2 in glioblastoma, we evaluated their baseline expression at the transcriptional and protein levels in the human glioblastoma cell lines U251 and U87MG.

**Figure 2:**
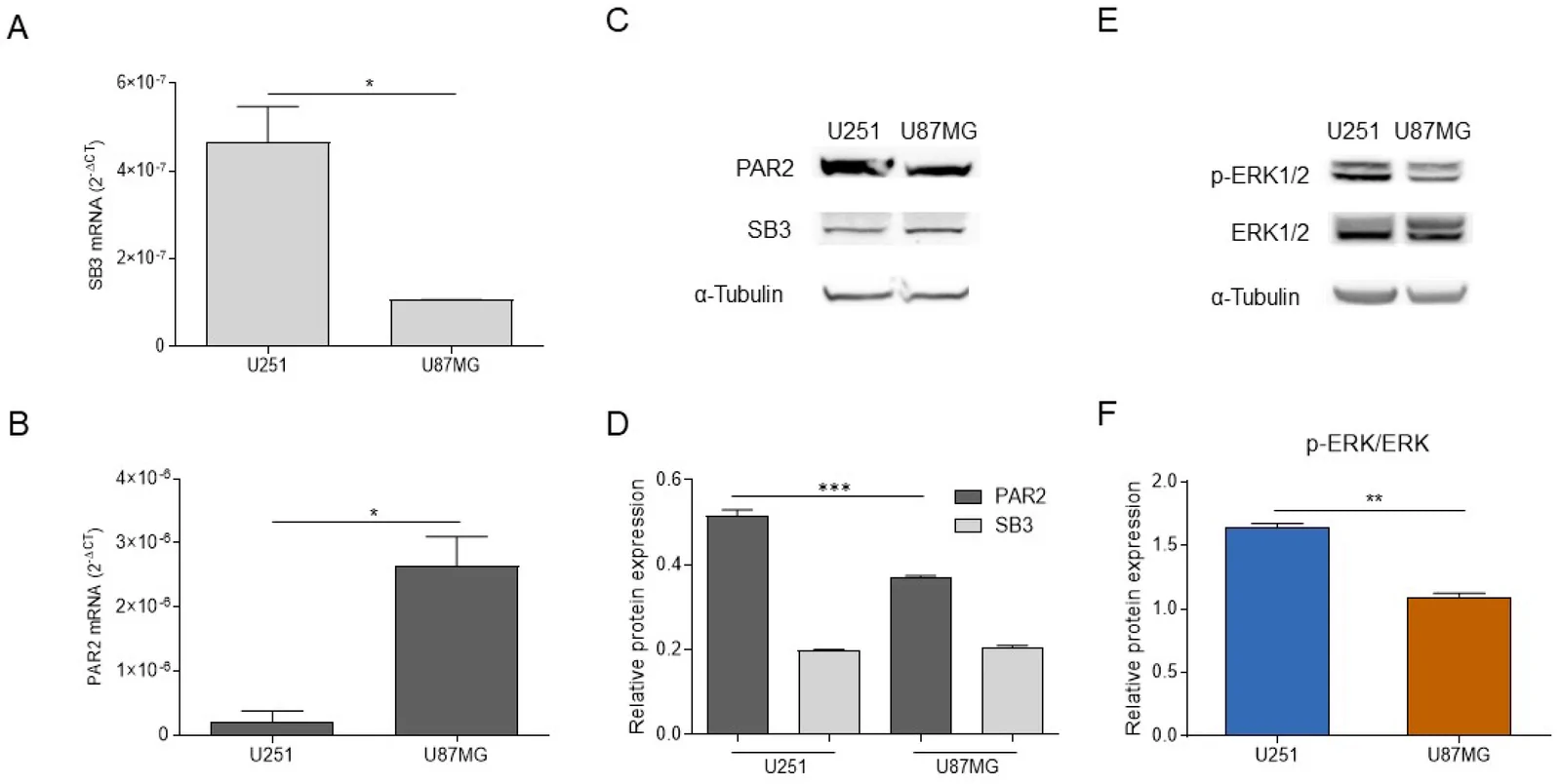
SerpinB3 and PAR2 expression in glioma cell lines. (**A**,**B**): SerpinB3 and PAR2 mRNA expression levels in U251 and U87MG glioblastoma cells, quantified by real-time PCR. (**C**,**D**): SerpinB3 and PAR2 protein expression assessed by Western blot analysis. (**E**,**F**): PAR2 activation evaluated by measuring the ratio of phosphorylated ERK to total ERK (p-ERK/total ERK) by Western blot. Images represent an example of three independent experiments. Quantitative densitometry data were normalized to the housekeeping protein α-tubulin. Statistical analysis was performed using unpaired Student’s *t*-test. * *p* < 0.05, ** *p* < 0.01, and *** *p* < 0.001.

At the transcriptional level, *SerpinB3* and *PAR2* mRNA displayed an inverse relationship. Specifically, U251 cells exhibited higher *SerpinB3* levels alongside lower *PAR2* mRNA expression (Figure 2A,B). However, at the protein level, both cell lines exhibited high PAR2 expression, with slightly, although significantly, higher levels in U251 cells, whereas SerpinB3 expression was comparable between the two cell lines (Figure 2C,D). The discrepancy between low *F2RL1* (*PAR2*) mRNA levels and high PAR2 protein abundance, particularly evident in U251 cells, could be explained by a possible homeostatic transcriptional negative feedback loop triggered by impaired protein turnover dependent on SerpinB3. This finding may reflect a failed cellular attempt to restore homeostatic balance by downregulating *F2RL1* mRNA, because of SerpinB3’s ability to interfere with the ubiquitin-proteasome system (UPS) [40].

Then, to evaluate the possible correlation between PAR2 expression and its activation in our models, we analyzed ERK1/2 phosphorylation (p-ERK1/2), a clear downstream marker of PAR2 activation [15,16,22,41,42,43]. Our data revealed that basal PAR2 protein levels positively correlate with ERK1/2 activation, being significantly higher in the U251 cell line. These results suggest that PAR2 signaling activation may represent a potential therapeutic target in glioblastoma.

### 2.3. Pharmacokinetics of 1-PPA in Serum and Organ Distribution

An analytical method, based on a mixed-mode (reverse-phase/cation-exchange mechanism) solid-phase extraction followed by LC-MS/MS with ESI in positive mode, was developed and applied for the determination of 1-PPA kinetics in serum and in different organs, with particular attention to brain distribution. Homogenate samples were obtained at different time points from drug-treated BALB/c wild-type male mice.

**Figure 3:**
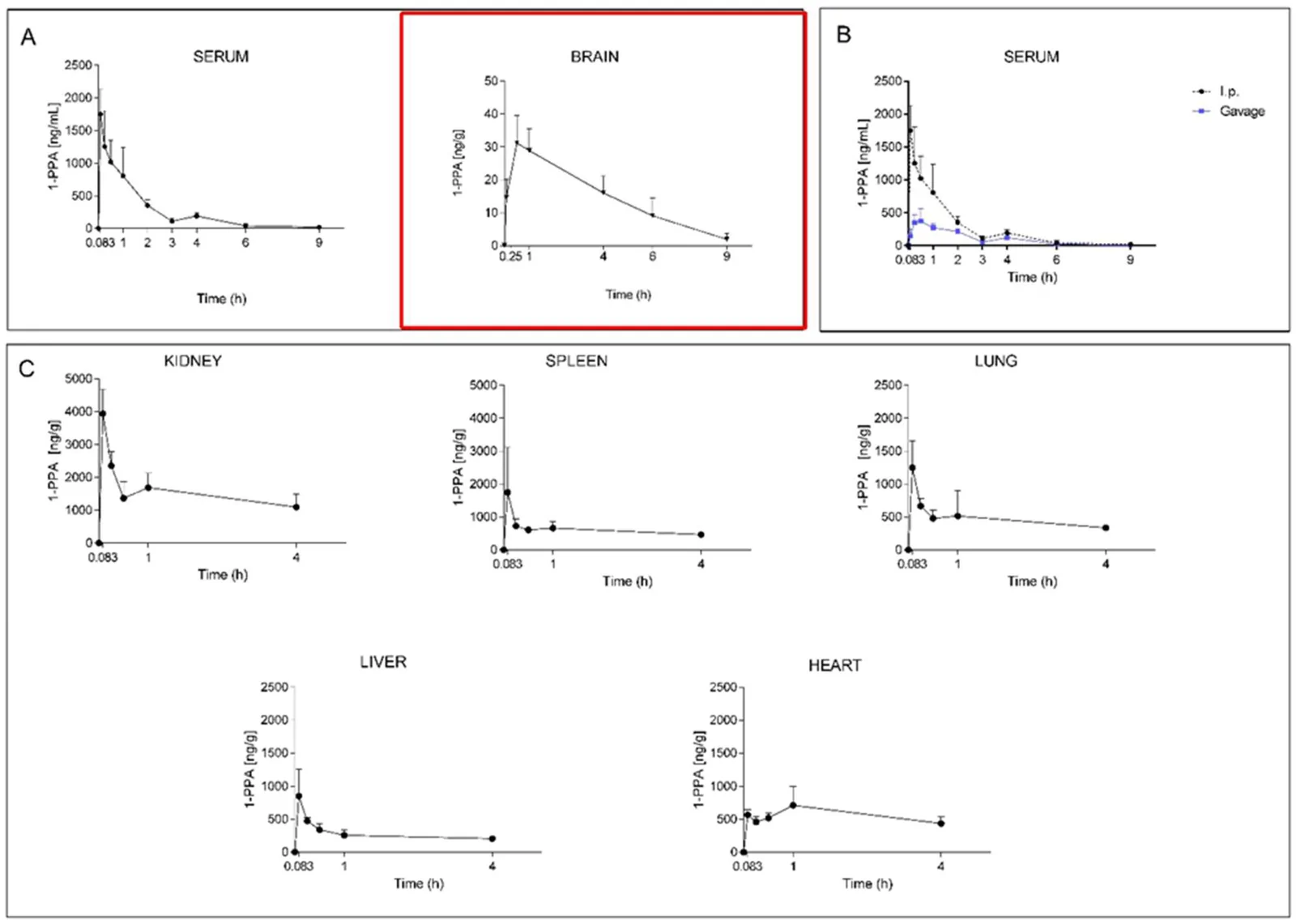
1-PPA profiles in serum and organ distribution. (**A**) Serum and brain 1-PPA concentration–time profiles following a single i.p. injection of 0.7 mg/kg 1-PPA to BALB/c wild-type male mice (n = at least 4 for each sampling time point). (**B**) Serum drug concentration–time profile following a single i.p. or p.o. (oral gavage) administration of 0.7 mg/kg 1-PPA to BALB/c wild-type male mice (n = 4 for each sampling time point). (**C**) Tissue concentration–time profiles of 1-PPA following a single i.p. injection of 0.7 mg/kg 1-PPA to BALB/c wild-type male mice (n = 4 for each sampling time point). Each value is the average concentration, and the bars represent standard deviations.

Figure 3A shows the serum and total brain drug concentration–time profiles after a single intra-peritoneal (i.p.) injection of 0.7 mg/kg 1-PPA, whereas Table S1 shows the mean serum concentration of 1-PPA, at each sampling timepoint, together with the standard deviation and the 95% confidence interval. The corresponding PK parameters, estimated by standard non-compartmental analysis with sparse sampling settings, are reported in Table 1.

The drug exhibited fast absorption from the peritoneal cavity (serum T_max_ = 0.083 h), as well as a non-negligible drug distribution in the brain, as the mean drug level in this organ, 0.5 h post-injection, was ~3.6% of that recorded at the same time point in serum (Table 1). 1-PPA peak time and maximum concentration were 0.5 h and 31 ± 3 µg/kg, respectively.

To verify oral absorption, 1-PPA was administered *per os* (p.o.) at a single dose of 0.7 mg/kg. Figure 3B shows the serum drug concentration–time profile after a single oral administration by gavage. The corresponding PK parameters, estimated by standard non-compartmental analysis, are reported in Table 2. T_max_, similar to the brain, was six times higher (0.5 vs. 0.083 h). The C_max_ and AUC_0–∞_ were roughly 5 and 2.5 times higher after i.p. compared to oral administration, respectively. The MRT value after oral administration was 1.3 times greater than that observed after i.p. injection. The bioavailability of the p.o. administration in relation to the i.p. injection was 40%, dosing 1-PPA 0.7 mg/kg for both routes of administration.

To study the distribution of 1-PPA in different organs, animals were sacrificed after 0.083, 0.25, 0.5, 1, and 4 h. Drug concentration–time profiles in the kidney, spleen, liver, lung, and heart of mice after a single i.p. injection of 0.7 mg/kg 1-PPA are shown in Figure 3C. Moreover, data on the tissue-to-serum ratio are shown in Table 3.

### 2.4. Blood–Brain Barrier Permeability of 1-PPA

The degree of BBB permeability (*LogBB* value) of 1-PPA was estimated using both a prediction model developed by Shaker et al. [44] (predicted *LogBB*) and in vivo data (in vivo *LogBB*). The predicted *LogBB* of 1-PPA, calculated by the free web software available at http://ssbio.cau.ac.kr/software/logbb_pred/ (accessed on 3 April 2026), was −0.59. For exploratory comparison, the predicted *LogBB* of TMZ, calculated using the same prediction model, was −0.51. TMZ is a well-established antitumor agent with activity in high-grade gliomas, including glioblastoma, due, at least in part, to its ability to overpass the BBB [45]. In vivo *LogBB* values for 1-PPA, calculated at 4, 6, and 9 h post i.p. injection were −1.07, −0.65, and −1, respectively.

### 2.5. Biological Effects of 1-PPA in Glioblastoma Cell Lines

Since our results suggested the capability of 1-PPA to possibly cross the BBB, at comparable levels of TMZ, the current gold-standard treatment in glioblastoma, we evaluated the potential antitumor efficacy of 1-PPA in glioblastoma preclinical models. To assess its effects in vitro, we analyzed effects of 1-PPA treatment on SerpinB3 and PAR2 expression in relation to cell proliferation and survival, migration, and invasion in human glioblastoma cells. The obtained results are displayed below.

(a) PPA inhibits the SerpinB3/PAR2 axis in glioblastoma cell lines.

**Figure 4:**
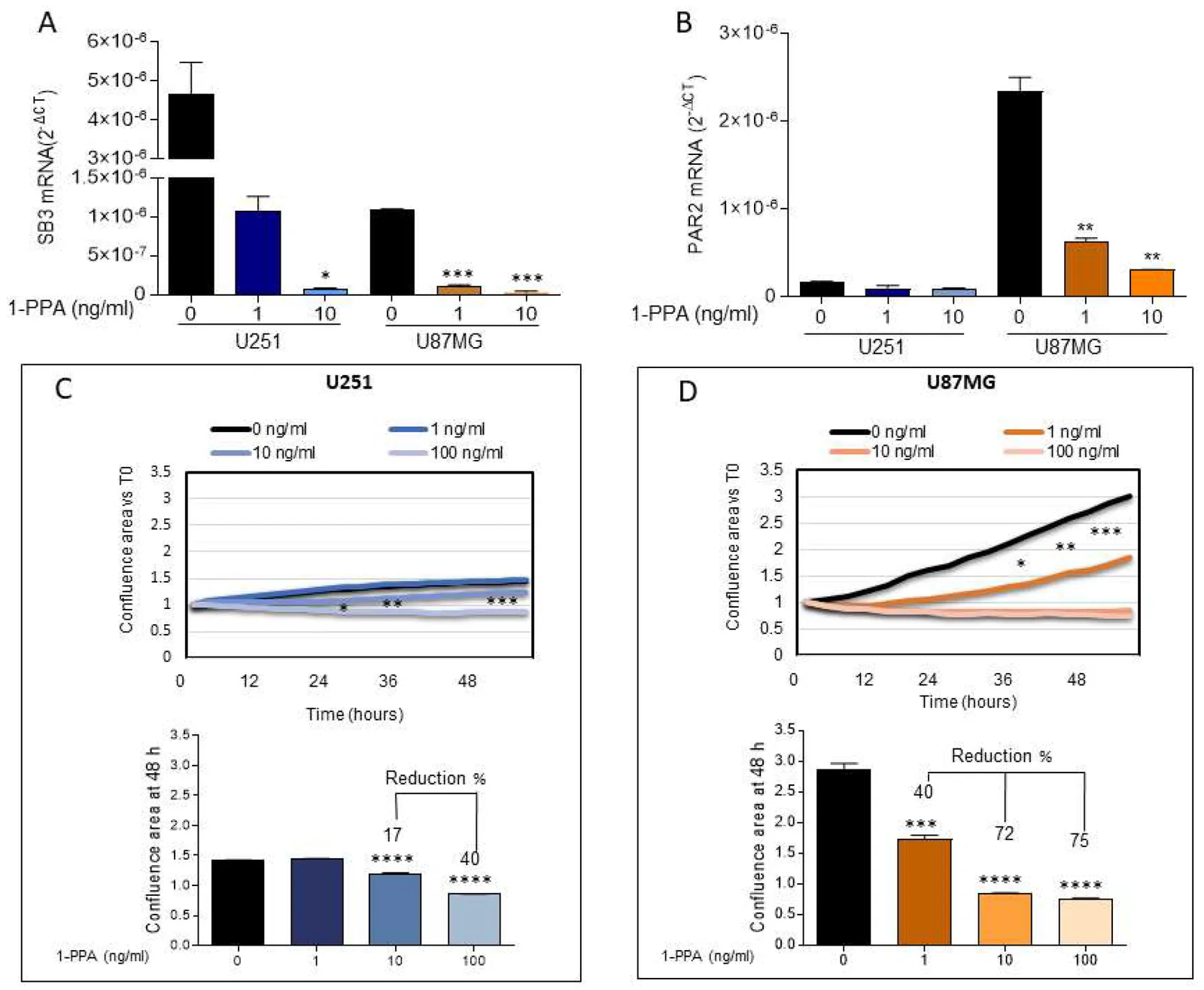
Effect of 1-PPA on SerpinB3/PAR2 expression and cell proliferation in glioblastoma cell lines. (**A**,**B**) SerpinB3 and PAR2 mRNA expression levels measured by real-time PCR after treatment with increasing concentrations of 1-PPA. (**C**,**D**) Effect of increasing doses of 1-PPA on real-time cell proliferation measured by the Incucyte S3 Live-Cell Analysis System. (**C**,**D**, higher panels) Cell proliferation evaluated over 48 h of treatment and expressed as confluence phase area relative to time 0 (t0). (**C**,**D**, lower panels) Histograms showing values of cell proliferation at 48 h of treatment. Data are presented as mean ± SEM. Statistical analysis was performed using two-way ANOVA followed by Bonferroni’s multiple comparisons test or unpaired Student’s *t*-test. * *p* < 0.05, ** *p* < 0.01, *** *p* < 0.001 and **** *p* < 0.0001.

Treatment with 1-PPA resulted in a concentration-dependent downregulation of SerpinB3 (Figure 4A) and PAR2 (Figure 4B) mRNA expression in both the glioblastoma cell lines analyzed.

(b) PPA inhibits cell proliferation in glioblastoma cell lines.

The proliferation of U251 (Figure 4C) and U87MG (Figure 4D) cell lines, as well as glioblastoma primary cells (Figure S2A,B), was inhibited by 1-PPA in a concentration-dependent manner. It is worth noting that the cell line more resistant to 1-PPA treatment was the U251, which displays, at baseline, the highest levels of PAR2 expression and ERK1/2 activation, as shown in Figure 2.

(c) PPA inhibits cell migration and invasion in glioblastoma cell lines.

One of the characteristics of glioblastoma is its ability to conduct tumoral cell infiltration and invasion, which supports the poor prognosis of this tumor [46,47]. To assess the antitumor efficacy of 1-PPA, we evaluated its capacity to inhibit cell migration and invasion in glioblastoma cell lines.

**Figure 5:**
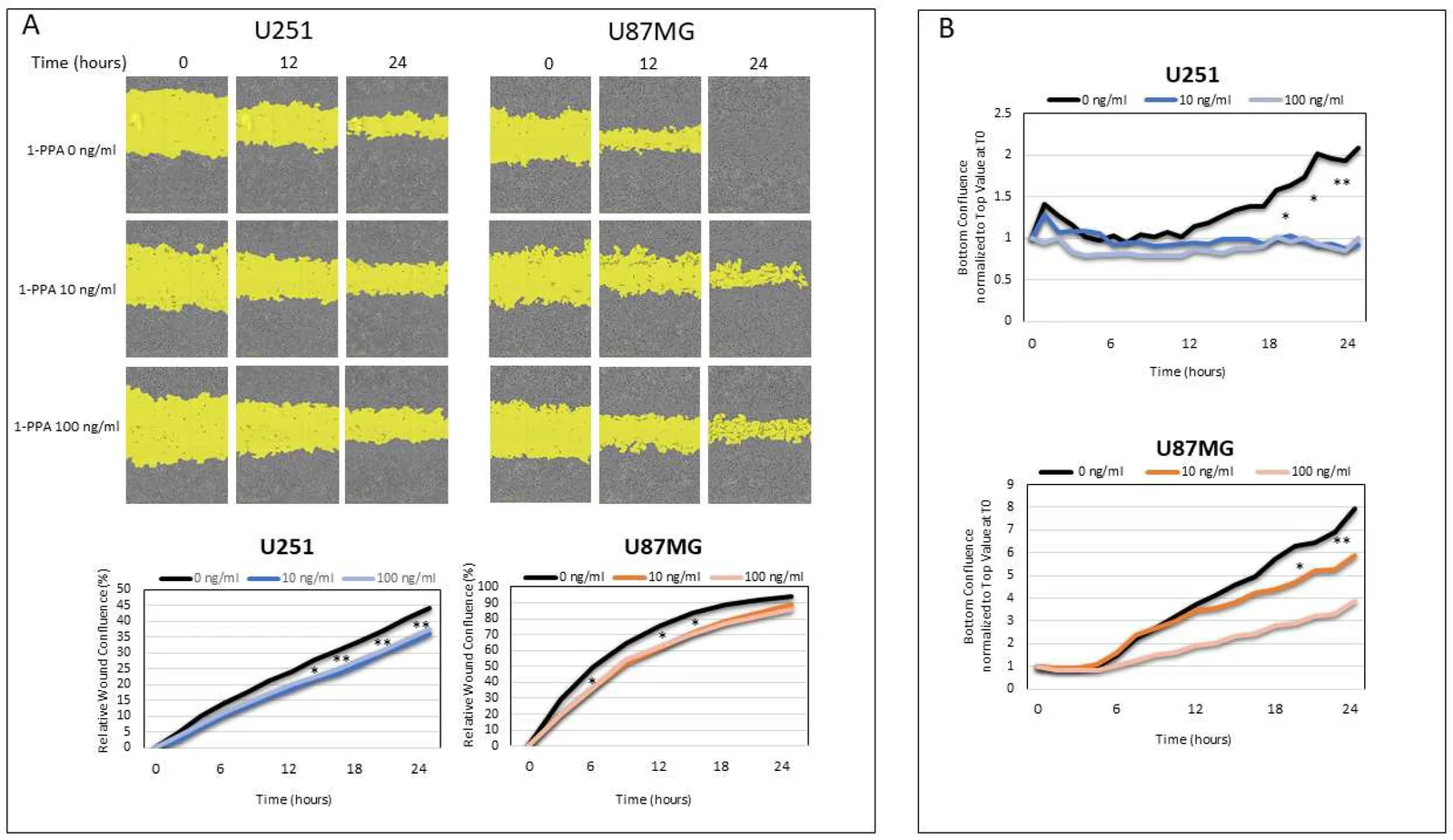
Inhibitory effects of 1-PPA on the migratory and invasive capacities of glioblastoma cells. (**A**) Collective cell migration was assessed using a scratch wound healing assay on U251 and U87MG cell lines. Representative phase-contrast images (**top**) showing the wound area masked in yellow at 0, 12, and 24 h of treatment with 0, 10, and 100 ng/mL of 1-PPA. Quantitative analysis (**bottom**) illustrates the kinetic changes in relative wound confluence (%) over a 24 h period. (**B**) Cell invasion capacity was evaluated using a real-time chemotactic Transwell assay in Incucyte^®^ ClearView 96-well plates under phase-contrast imaging. Graphs represent the time-course quantification of cell invasion, expressed as bottom confluence normalized to the top value at time zero, for U251 (**top**) and U87MG (**bottom**) cells treated with different concentrations of 1-PPA (0, 10, and 100 ng/mL). Data are expressed as mean ± SEM. Statistical significance was determined by two-way ANOVA followed by Bonferroni’s multiple comparisons test. * *p* < 0.05; ** *p* < 0.01 compared to the respective control group (0 ng/mL).

In the scratch wound healing assay, performed using the Incucyte^®^ S3 live-cell imaging system, we show that the inhibitory effect of 1-PPA is statistically significant in both cell lines. Specifically, a significant inhibition of relative wound confluence was observed starting from the 14th hour of treatment for the U251 cell line (*p* < 0.05; * *p* < 0.01) and from the 6th hour for the U87MG cell line (*p* < 0.05) (Figure 5A).

The impact of 1-PPA on the invasive capacity of glioblastoma cells was evaluated under phase contrast using Incucyte^®^ ClearView 96-well plates (Figure 5B). Statistical analysis demonstrated a significant inhibition of cell invasion, expressed as bottom confluence normalized to the top value at time zero, starting from the 20th hour of treatment in both cell lines. Specifically, U251 cells showed a dramatic and rapid suppression of invasive potential at both 10 ng/mL and 100 ng/mL, with bottom confluence remaining close to baseline levels throughout the 24 h period. For U87MG cells, 1-PPA exerted a clear, dose-dependent inhibitory effect, where the highest concentration (100 ng/mL) led to a robust reduction in cell invasion compared to the control group (** *p* < 0.01). Indeed, U87MG cells migrate predominantly as individual cells with faster kinetics toward the chemoattractant, a process driven by their elevated levels of MMPs.

Together, these data indicate that 1-PPA effectively inhibits both collective and single-cell infiltration mechanisms, regardless of the specific phenotypic background of the glioblastoma cells analyzed.

(d) 1-PPA sensitizes glioblastoma cell lines to apoptosis.

**Figure 6:**
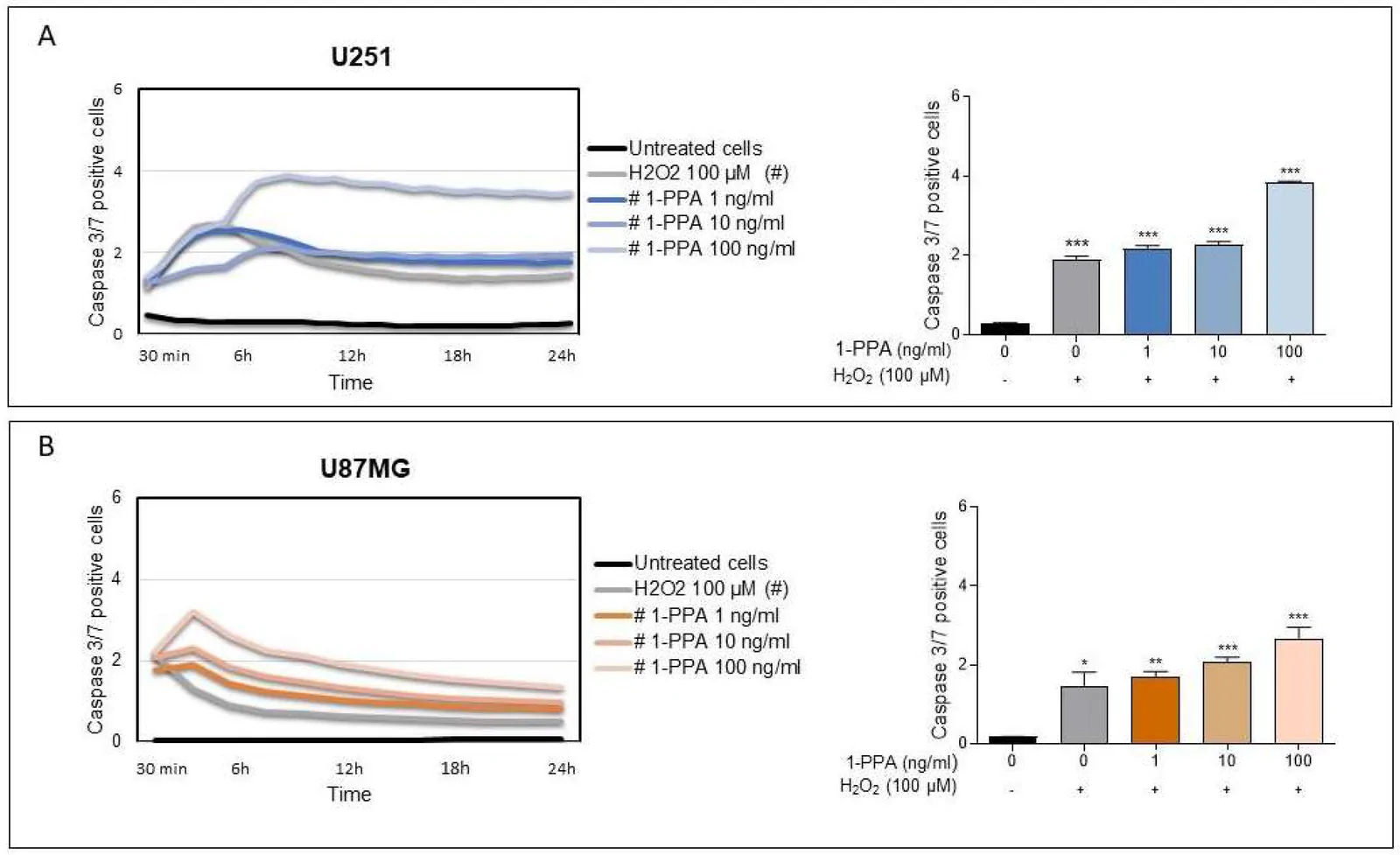
Effect of 1-PPA on glioblastoma cell apoptosis. Apoptosis was assessed by monitoring caspase-3/7 activity using the Incucyte^®^ Live-Cell Analysis System S3. Cells were treated with 100 µM H_2_O_2_ (#) to induce apoptotic cell death, followed by treatment with increasing concentrations of 1-PPA (+ and – indicate H_2_O_2_ addition). Real-time green fluorescence, indicative of caspase-3/7 activation, quantified over 24 h after 30 min of prewarming the plate at 37 °C, is represented for U251 cells (left panel, (**A**)) and U87MG cells (left panel, (**B**)). Graphical representation of green area confluence in the absence or presence of 1-PPA (10–100 ng/mL) at the peak of the highest apoptotic response is represented for U251 cells (right panel, (**A**)) and U87MG cells (right panel, (**B**)). Data are presented as mean ± SEM. Statistical analysis was performed using two-way ANOVA followed by Bonferroni’s multiple comparisons test or an unpaired Student’s *t*-test. * p < 0.05, ** *p* < 0.01, and *** *p* < 0.001.

Resistance to apoptosis remains a critical challenge in identifying novel therapeutic targets for aggressive tumors, particularly for glioblastoma [48]. To address this issue, we examined the apoptotic response of our glioblastoma cell lines to 1-PPA using a standard pro-apoptotic stimulus, hydrogen peroxide (H_2_O_2_) at 100 μM, which was chosen from preliminary experiments as the most suitable concentration for our experimental setting. As shown in Figure 6, glioblastoma cell lines displayed a poor basal apoptotic response to H_2_O_2_, likely reflecting their high resistance to several pro-apoptotic stimuli, maybe through expression of SerpinB3 and PAR2 activation [14,20,49,50,51]. Importantly, 1-PPA treatment determined a dose-dependent increase in apoptotic cell death in all tested cell lines (Figure 6A,B), accompanied by a marked decrease in both PAR2 and SerpinB3 expression (Figure 4A,B). This downregulation potentially suppresses their anti-apoptotic functions, thereby facilitating the induction of apoptosis. The magnitude and kinetics of the caspase-3/7-associated response moderately differed between U251 and U87MG cells, consistent with the distinct biological characteristics of the two cell lines. Following the initial increase, the caspase-3/7-associated signal reached a plateau after approximately 8–10 h (Figure 6A,B, left panels), in line with the early expected effect of this pro-apoptotic stimulus.

### 2.6. Combined Effect of 1-PPA and Temozolomide on U251 Cell Proliferation

**Figure 7:**
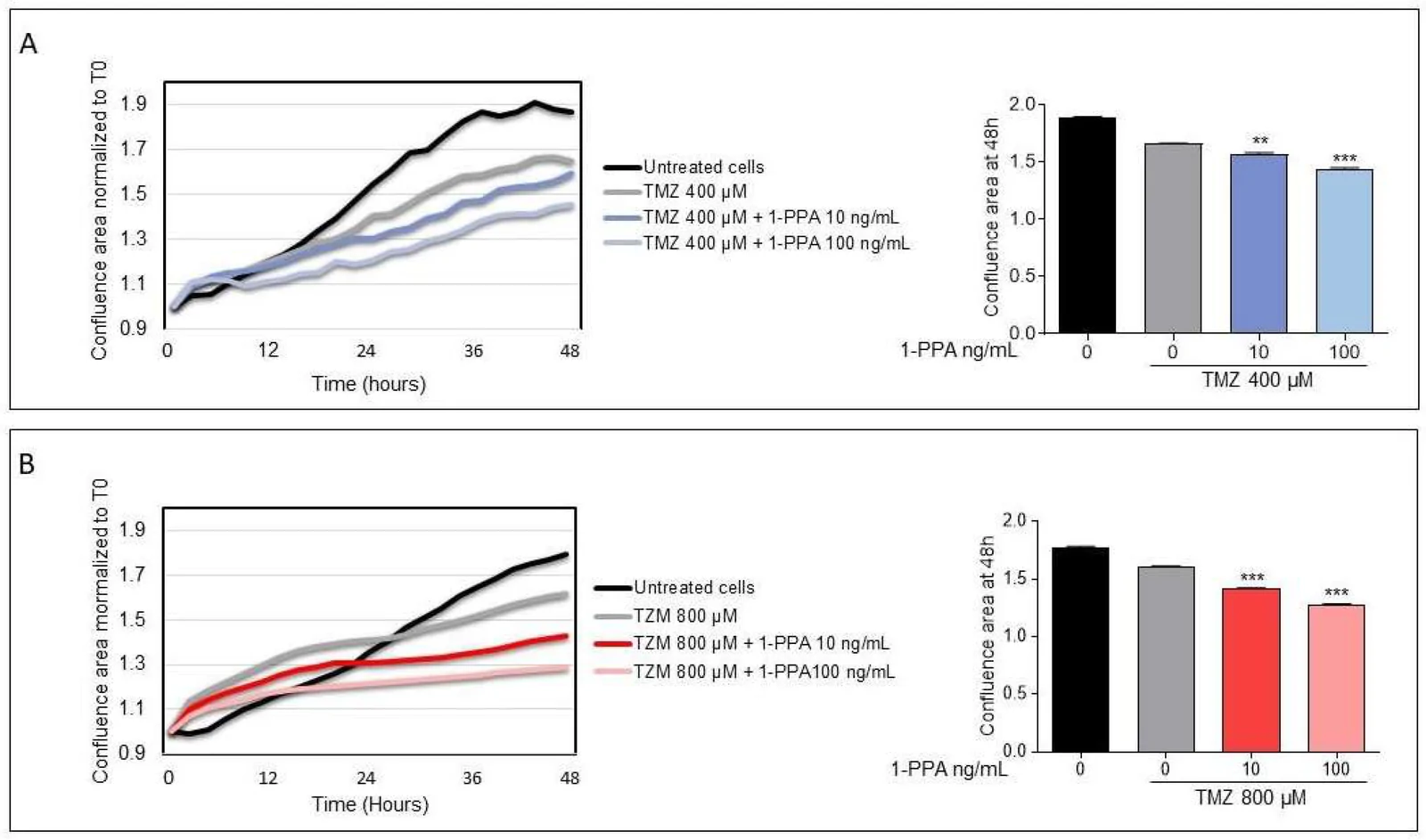
Combined effect of 1-PPA and temozolomide (TMZ) on U251 cell proliferation. The effect of increasing concentrations of 1-PPA in combination with TMZ (400 µM in left panel, A; 800 µM in left panel, **B**) on cell proliferation evaluated over 48 h of treatment and expressed as confluence phase area relative to time 0 (T0) (left panel, (**A**,**B**)). Histograms show values of cell proliferation at 48 h of treatment (right panel, (**A**,**B**)). Data are presented as mean ± SEM. The statistical significance reported compares the response of the combined treatment with that of TMZ alone. Statistical analysis was performed using two-way ANOVA followed by Bonferroni’s multiple comparisons test and an unpaired Student’s *t*-test. ** *p* < 0.01; *** *p* < 0.001.

Based on the suggested role of the SerpinB3/PAR2 axis in glioblastoma [14,20], to investigate whether 1-PPA could sensitize glioblastoma cells to standard chemotherapy, as suggested by PAR2 modulation in other contexts [51], we evaluated the synergistic potential of 1-PPA in combination with TMZ. The U251 cell line was selected for these assays due to its high basal levels of PAR2 expression/activation. Our results demonstrate that the addition of 1-PPA significantly enhanced the antiproliferative activity of TMZ in a concentration-dependent manner. Specifically, co-treatment with 1-PPA and TMZ, compared to TMZ alone, resulted in a 12% and 20% reduction in cell proliferation at TMZ concentrations of 400 μM and 800 μM, respectively, in the presence of 1-PPA at 100 ng/mL (Figure 7). To corroborate these results, we also tested the combined effect of 1-PPA and TMZ in two different primary glioblastoma cultures. A sulforhodamine B-based viability assay confirmed a slightly, although significant, additive antiproliferative effect when 1-PPA and TMZ were combined, compared to single treatments (Figure S2C).

These results indicate that 1-PPA improves the therapeutic response of TMZ when they are combined, leading to a relevant reduction in cell proliferation compared to control cells.

## 3. Discussion

Glioblastoma remains one of the most challenging and devastating malignancies in neuro-oncology. Despite advances in clinical management, the prognosis for patients remains extremely poor [1,4,5]. The aggressive nature of glioblastoma depends on its rapid proliferation, diffuse infiltration of surrounding brain tissue, and marked intratumoral heterogeneity, which significantly affect treatment efficacy. In addition, the BBB constitutes a further barrier to drug delivery, restricting the therapeutic potential of many promising agents [52]. The current standard of care offers only modest survival benefits, allowing for almost inevitable recurrence, which often displays increased resistance to further administered therapies [45]. These aspects underscore the urgent need for novel treatment strategies that can overcome these biological barriers and target the fundamental drivers of glioblastoma aggressiveness.

In this context, our study provides evidence for the potential of 1-PPA as a novel therapeutic agent for glioblastoma. Our findings demonstrate that 1-PPA achieves measurable brain exposure, possesses favorable pharmacokinetics, and exerts antitumor effects in vitro by inhibiting the PAR2/SerpinB3 signaling axis in glioblastoma cells.

A critical challenge in developing effective treatments for glioblastoma is achieving sufficient drug concentrations within the brain [9]. Our pharmacokinetic analysis revealed that 1-PPA exhibits rapid absorption following i.p. administration, with detectable levels in both serum and brain tissue within minutes of injection. Importantly, 1-PPA demonstrated brain distribution, with concentrations reaching approximately 3.6% of serum levels at 30 min post-injection. This brain-to-serum ratio is comparable to or exceeds that of several currently approved drugs for central nervous system (CNS) indications [53], suggesting that 1-PPA is capable of reaching measurable concentrations within the brain compartment.

The extended retention time of 1-PPA in brain tissue (MRT brain = 3.2 h vs. MRT serum = 1.8 h) is particularly noteworthy, as it indicates the potential for sustained therapeutic effects with less frequent dosing. The observed fast absorption of 1-PPA is not surprising, as compounds formulated as aqueous solutions typically reach an early and high plasma/serum peak concentration after i.p. injection [52]. Furthermore, the bioavailability of 40% compared to i.p. administration supports the use of the oral route for chronic treatment regimens, potentially improving patient compliance and quality of life compared to parenteral therapies. Importantly, our previous studies also disclosed that 1-PPA treatment is not associated with cell or organ toxicity in both in vitro and in vivo models [15,24].

Our in silico and in vivo *LogBB* calculations suggest that 1-PPA has the potential for measurable brain exposure. The predicted *LogBB* value (−0.59) and experimentally derived *LogBB* values (ranging from −1.07 to −0.65) are consistent with limited but detectable BBB penetration. However, because brain concentrations were determined in non-perfused whole-brain homogenates and were not corrected for residual intravascular blood, these findings should be interpreted as evidence of brain exposure rather than definitive proof of drug distribution to brain parenchyma. Furthermore, unbound brain-to-plasma ratios, brain-to-plasma partition coefficients, and complementary approaches such as capillary depletion or microdialysis were not assessed in the present study and may provide a more rigorous evaluation of parenchymal drug exposure.

For exploratory comparison, the predicted *LogBB* of TMZ, calculated using the same in silico model, was −0.51; however, this comparison is preliminary and insufficient alone to establish comparable BBB permeability. It should be emphasized that *LogBB* values, whether predicted or experimentally determined, are parameters that reflect the potential for CNS exposure but do not directly demonstrate pharmacologically active drug concentrations within the brain parenchyma or predict therapeutic efficacy. Therefore, further pharmacokinetic and pharmacodynamic studies are warranted to assess actual CNS drug levels and the potential therapeutic relevance of 1-PPA in glioblastoma. Direct comparisons between brain concentrations achieved in vivo and concentrations active in vitro are complicated by factors such as protein binding, tissue distribution, uptake by target (tumor) cells, and the lack of information on unbound brain concentrations. Accordingly, the present data do not allow a rigorous assessment of the relationship between brain exposure and the concentration-response effects observed in vitro.

Our in vitro studies provide evidence that 1-PPA may exert multifaceted antitumor effects in glioblastoma cells. This compound was indeed able to inhibit cell proliferation, since 1-PPA treatment resulted in a concentration-dependent reduction in cell growth in different glioblastoma cell lines and primary cultures. These antiproliferative effects support the concept that inhibition of the PAR2/SerpinB3 axis may be sufficient to directly impair glioblastoma cell growth. In this context, PAR2 has been reported to regulate VEGF production through the ERK1/2 pathway in glioblastoma cells [17]. Moreover, PAR2 silencing has already been correlated with ERK1/2 inhibition in several contexts [41,54,55], further supporting the relevance of these results. Although co-treatment with TMZ further enhanced the antiproliferative response of TMZ, the general magnitude of the 1-PPA-dependent sensitizing effect was relatively modest. Nevertheless, these results collectively suggest that 1-PPA may represent a promising therapeutic candidate both as a standalone treatment and as a part of combination strategies with chemotherapy.

Intriguingly, 1-PPA was able to significantly affect cell migration and invasion, reducing both collective and individual cell motility in glioblastoma cells, regardless of their baseline invasive phenotype. This is particularly important given that infiltrative growth is a hallmark of glioblastoma and a major contributor to treatment escape and recurrence [46,56,57,58].

Furthermore, treatment with 1-PPA enhanced the apoptotic sensitivity of glioblastoma cells, documented by increased susceptibility to oxidative stress-induced apoptosis, and by real-time monitoring of executioner caspase-3/7 activation. Since caspase-3/7 activation represents a key event in the execution phase of apoptosis, this approach provides a functional measure of apoptotic progression in living cells [59]. Supporting this result, inhibition of the PAR2/ERK1/2 signaling axis has recently been correlated to apoptosis sensitization in colorectal cancer cells [41]. These findings suggest that 1-PPA promotes engagement of the apoptotic machinery and may contribute to mitigating the intrinsic resistance to apoptosis of this tumor [8,14].

Provided analyses demonstrate that the 1-PPA target, PAR2, is significantly upregulated in glioblastoma, and clearly associated with tumor aggressiveness. This is consistent with previous reports demonstrating the overexpression of PAR2 in multiple human malignancies, including colorectal, ovarian, lung, pancreatic, and other cancers, including glioblastoma [14,17], where it supports proliferation, invasion, angiogenesis, stemness, and therapeutic resistance [12,60,61,62,63,64]. Along this line, PAR2 expression was significantly reduced in glioblastomas belonging to the proneural subtype, generally characterized by lower invasiveness and a more favorable biological profile than mesenchymal tumors [32,33]. Intriguingly, PAR2 expression displayed no association with IDH mutation status [35], suggesting that its expression may primarily reflect tumor aggressiveness rather than being dependent on specific genetic alterations, underlining its potential as a broad target in glioblastoma tumors.

To the best of our knowledge, no systematic selectivity profiling of 1-PPA against other molecular targets has been performed. Our previous study demonstrated that 1-PPA binds an allosteric pocket of PAR2 supported by CETSA, molecular docking, and molecular dynamics analyses [22]. Interestingly, this allosteric pocket is highly conserved among members of the PAR receptor family, suggesting that potential off-target activity, if present, is most likely restricted to other PAR receptors. However, this hypothesis has not yet been experimentally validated, and comprehensive target deconvolution studies will be required to fully define the selectivity profile of 1-PPA.

In conclusion, our data support a model in which 1-PPA attenuates the PAR2/SerpinB3 signaling axis. Indeed, treatment with 1-PPA led to downregulation of both PAR2 and SerpinB3 mRNA expression in glioblastoma cells, suggesting that the effects of 1-PPA may rely on functional PAR2 expression and the interference with a pro-oncogenic circuit regulated by the activity of PAR2/SerpinB3 signaling.

## 4. Materials and Methods

### 4.1. Cell Culture and Treatments

Human glioblastoma cell lines U251 and U87MG were purchased from Merck (Cat. No. 09063001, Darmstadt, Germany) and ATCC (Cat. No. HTB-14, Manassas, VA, USA), respectively. Cells were routinely tested for the presence of mycoplasma and were used at low passage after receipt from the suppliers. Cells were cultured in Dulbecco’s Modified Eagle Medium (DMEM; Merck, Darmstadt, Germany) supplemented with 10% fetal bovine serum (FBS), 100 U/mL penicillin, 100 μg/mL streptomycin, and 2 mM L-glutamine (all from Merck, Darmstadt, Germany). Cultures were maintained at 37 °C in a humidified incubator with 5% CO_2_. Primary glioblastoma cells, obtained from surgical resection specimens of patients with glioblastoma, were isolated and cultured according to previously described methods [65,66], as detailed in the Supplementary Material (Materials and Methods Section).

For treatment experiments, cells were exposed to serial dilutions (1, 10, and 100 ng/mL) of 1-PPA (Merck/Sigma-Aldrich, St. Louis, MO, USA) alone or in combination with TMZ (400–800 μM, Selleck Chemicals, Houston, TX, USA) dissolved in DMSO. Control cells received the vehicle alone at equivalent dilution (final concentration < 1% (*v*/*v*)). Treatments were performed for the indicated time points, according to the specific experimental assay.

### 4.2. Biological Effects of 1-PPA Treatment

Live-Cell Imaging Assays. Live-cell imaging analyses were performed using the Incucyte S3 Live-Cell Analysis System (Sartorius AG, Göttingen, Germany).

Cell Proliferation. Cells were seeded at 1 × 10^4^ cells per well in 96-well plates and allowed to adhere for 18–24 h before treatment with 1-PPA at the indicated concentrations. Each experimental condition was performed in octuplicate. Phase-contrast images were acquired every 2 h for 48 h using a 10x objective. Cell proliferation was quantified using Incucyte software (2024A) by measuring cell confluence over time and reported as fold-change relative to baseline (t0).

In some experiments involving primary glioblastoma cells, sulforhodamine B (SRB) staining was used as an orthogonal method to measure proliferative inhibition. Briefly, cells were seeded and, after 24 h, treated with 1-PPA (100 ng/mL), TMZ (400 μM), or a combination of them both for 72 h. Then, cells were fixed in 10% trichloroacetic acid (AppliChem GmbH, Darmstadt, Germany) for at least 1 h at 4 °C, washed in H_2_O, dried, stained with a 0.04% SRB solution (Thermo Fisher Scientific, Waltham, MA, USA) in 1% acetic acid for 1 h, and then rinsed in 1% acetic acid. SRB was then solubilized in 10 mM Tris-base, and absorbance was detected at 510 nm through a Spark10M fluorimeter (Tecan, Mannedorf, Switzerland).

Cell Migration (Scratch Assay). For migration assays, U251 and U87MG cells were seeded at 5 × 10^5^ cells per well in 96-well ImageLock plates (Sartorius AG, Göttingen, Germany) and grown to near confluence. Uniform wounds were generated using the WoundMaker device (Sartorius AG). Cells were then treated with 1-PPA (range: 10–100 ng/mL). The culture plate was placed in an Incucyte S3 instrument (Sartorius AG, Goettingen, Germany) and kept in a dedicated incubator. Automated phase-contrast images were acquired every 3 h for up to 24 h using a 10× objective within the Incucyte^®^ Live-Cell Imaging System (Sartorius AG). Quantitative kinetic analysis of cell motility was performed using the Incucyte^®^ 2024A software based on two distinct metrics; in particular, Relative Wound Density (RWD) and Wound Width (µm). RWD quantifies the spatial cell density within the wounded area relative to the cell density of the surrounding monolayer, effectively neutralizing the confounding effects of cell proliferation. Wound Width measures the average distance (µm) between the advancing edges of the cell monolayer over time. Together, these complementary parameters provide a robust kinetic assessment of cell migration.

Cell Invasion (chemotaxis-driven). Cell invasion under a chemotactic gradient was evaluated using Incucyte^®^ ClearView 96-well chemotaxis plates (Sartorius AG, Göttingen, Germany). Briefly, 1 × 10^3^ cells per well were embedded in 40 μL MaxGel extracellular matrix and combined with 20 μL of 3x concentrated 1-PPA to achieve the desired final concentration (10–100 ng/mL), before seeding into the upper chamber (total volume 60 μL). After 30 min at 37 °C, to allow matrix polymerization, 200 μL of chemoattractant-containing or control medium were added to the lower reservoir to establish a gradient. Automated imaging using a 10X objective was performed every hour for 24 h using the Incucyte^®^ Live-Cell Analysis System. Data analysis was executed via the Incucyte^®^ Chemotaxis Analysis Software (2024A). Proliferation and seeding biases were neutralized by normalizing the lower chamber cell confluence to the initial cell count in the upper chamber at time zero (initial top value normalization).

Apoptosis Assay. Apoptosis was assessed by real-time detection of caspase-3/7 activation using the Incucyte^®^ Live-Cell Analysis System S3 (Sartorius AG, Göttingen, Germany). Briefly, 1 × 10^4^ cells per well were seeded in 96-well plates and allowed to adhere for 18–24 h. Cells were then exposed to hydrogen peroxide (100 μM) to induce apoptosis and treated with increasing concentrations (1–100 ng/mL) of 1-PPA. The Incucyte^®^ caspase-3/7 Green Dye (Sartorius AG, Göttingen, Germany) was added at a final dilution of 1:1000. This cell-permeable fluorogenic substrate contains the DEVD peptide sequence, which is selectively cleaved by activated caspase-3 and -7 during apoptosis. Cleavage releases a DNA-binding fluorophore that selectively labels the nuclei of apoptotic cells, enabling quantitative real-time imaging of caspase-3/7-positive cells in living cultures [67]. Before the first scan, the plate was warmed at 37 °C for 30 min. Images were acquired every 2 h for 48 h using a 10x objective. Apoptosis was quantified as green object count per well at each time point and analyzed using Incucyte software (version 2024A).

### 4.3. Western Blotting

The expression levels of total-ERK1/2 and phospho-ERK1/2 were analyzed in U251 and U87MG glioblastoma cell lines by Western blotting. Briefly, total protein extracts were prepared using RIPA buffer (Merck Millipore, Burlington, MA, USA) supplemented with protease and phosphatase inhibitor cocktails (Thermo Fisher Scientific/Pierce, Rockford, IL, USA). Protein concentration was determined using the BCA Protein Assay Kit (Thermo Fisher Scientific/Pierce, Rockford, IL, USA). Equal amounts of protein (30 µg) were separated on 4–12% Bis-Tris Mini-PROTEAN gels (Thermo Fisher Scientific/Invitrogen, Carlsbad, CA, USA) and transferred onto nitrocellulose membranes using the Power Blotter XL System (Thermo Fisher Scientific/Invitrogen, Carlsbad, CA, USA). Membranes were blocked in 5% non-fat dry milk in TBS-T for 1 h at room temperature and incubated overnight at 4 °C with primary antibodies against SerpinB3, PAR2, phospho, and total ERK1/2 according to the manufacturers’ instructions (details on antibody suppliers are reported in Table S2).

After washing, membranes were incubated with horseradish peroxidase (HRP)-conjugated anti-mouse secondary antibody (KPL, Gaithersburg, MD, USA) or anti-rabbit secondary antibody (Sigma-Aldrich (Merck), St. Louis, MO, USA). Protein bands were visualized using enhanced chemiluminescence substrate (Euroclone S.p.A., Milano, Italy) and acquired with the Alliance Q9 Atom imaging system (Uvitec, Cambridge, UK). Densitometric analysis was performed using the manufacturer’s software. All Western blot experiments were independently repeated three times using distinct biological samples, and protein expression levels were normalized to α-tubulin (Proteintech, Rosemont, IL, USA) as loading control.

### 4.4. Real-Time PCR

Total RNA was extracted from glioblastoma cells using Trizol Reagent (Invitrogen, Carlsbad, CA, USA) according to the manufacturer’s instructions. After determination of the purity and the integrity of total RNA, complementary DNA synthesis was carried out from 1 μg of RNA using LunaScript RT SuperMix (New England BioLabs, Ipswich, MA, USA). Quantitative real-time PCR reactions (RT-PCR) were performed according to the Luna Universal qPCR master Mix (New England Biolabs, Ipswich, MA, USA) protocol, using the CFX96 Real-Time instrument (Bio-Rad Laboratories Inc., Hercules, CA, USA). The relative gene expression was generated for each sample by calculating 2^−ΔCt^ [68]. Primers sequences used in the study are reported in Supplementary Table S3.

### 4.5. In Vivo Pharmacokinetics and Organ Distribution

Pharmacokinetics and biodistribution of 1-PPA were evaluated in male BALB/c mice (10–12 weeks old; 28.7 ± 3.4 g), housed under controlled environmental conditions (12 h light/dark cycle; 21 ± 1 °C). 1-PPA (0.7 mg/kg in sterile 0.9% sodium chloride) was administered either intraperitoneally (i.p.; 4 animals per time point) or orally (p.o.; 4 animals per time point). Randomization was performed using a computer-generated random sequence created with Microsoft Excel (RAND function) (Office 2024). As this represents a preliminary pharmacokinetic assessment, male animals were selected to obtain an initial estimation of PK parameters with reduced biological variability before proceeding to studies inclusive of both sexes.

Blood samples were collected for up to 540 min post-dosing, centrifuged, and serum stored at −80 °C. For tissue distribution studies, mice received i.p. 1-PPA and organs (brain, kidney, spleen, lung, liver, and heart) were collected at selected time points, snap-frozen in liquid nitrogen, and stored at −80 °C. Animals were not transcardially perfused before tissue collection. Following euthanasia, whole brains were rapidly excised, weighed, snap-frozen, and stored at −80 °C until LC-MS/MS analysis. Consequently, brain concentrations were determined in non-perfused whole-brain homogenates and were not corrected for residual intravascular blood. To further characterize 1-PPA brain distribution, additional sampling was performed at 6 and 9 h.

All procedures were conducted under sevoflurane anesthesia, and the protocol was approved (11 November 2024) by the University of Padua Animal Welfare Committee and the Italian Ministry of Health (n° 1059/2024-PR), in compliance with EU regulations.

### 4.6. LC-MS/MS Quantification of 1-PPA

**Sample Preparation.** 1-PPA was extracted from serum and tissue homogenates using Oasis PRiME MCX solid-phase extraction cartridges (Waters Corporation, Milford, MA, USA). Serum (50 μL) was acidified with 4% H_3_PO_4_ and processed through conditioned cartridges. Elution was performed with ammonium hydroxide in methanol, followed by evaporation and reconstitution prior to analysis. Tissue homogenates were prepared in water, centrifuged at 13,684× *g* for 20 min at 4 °C, and the resulting supernatants were processed as described for serum.

**Method Validation.** Calibration curves (1–500 ng/mL) were prepared in blank samples of serum and brain homogenates. Linearity was confirmed (R^2^ ≥ 0.99), and the lower limit of quantification was established at 1 ng/mL (precision ≤ 20%, accuracy 80–120%). Extraction recovery exceeded 95% for all matrices.

**Chromatographic and Mass Spectrometric Conditions.** Chromatographic separation was achieved using an Accela 600 HPLC system coupled to an LTQ XL ion trap mass spectrometer (Thermo Fisher Scientific, San Jose, CA, USA). Detection was performed in positive electrospray ionization mode by monitoring the transition m/z 159 → 98. The retention time of 1-PPA was 2.04 min. Data acquisition and processing were performed using Xcalibur software (v 4.8).

**Blood–Brain Barrier Permeability.** BBB permeability was assessed by calculating experimental *LogBB* values:

(1) $$LogBB=Log\left(\frac{C_{Brain}}{C_{Blood}}\right).$$

This approach has been widely used in the pharmaceutical industry to support drug discovery programs [69]. Predicted *LogBB* values for 1-PPA and TMZ were obtained using a LightGBM-based in silico regression model. Compounds with *LogBB* ≥ −1 were considered BBB-permeable [44].

Further methodological details and extended pharmacokinetic and organ distribution analyses are reported in the Supplementary Material (Materials and Methods Section).

### 4.7. Pharmacokinetic Analysis

At each sampling time point, a distinct group of 4 p.o.- or 4 i.p.-treated animals was used (destructive/serial sacrifice design), resulting in a sparse sampling approach.

Pharmacokinetic (PK) parameters (C_max_, T_max_, AUC, and terminal half-life (t_1/2_)) in serum and brain, following i.p. administration, or in serum following p.o. administration, were calculated using Phoenix WinNonlin software version 8.3.5.349 (Certara, Princeton, NJ, USA). A non-compartmental analysis (NCA) model with sparse sampling settings was applied, implementing the Bailer method for the estimation of the area under the serum concentration–time curve (AUC) and its associated standard error (SEM).

### 4.8. Statistical Analysis

Quantitative proliferation (phase confluence area), migration (kinetic data for Relative Wound Density (RWD)), and invasion (bottom phase confluence area) were automatically generated by the Incucyte^®^ 2024A software. Data points are expressed as mean ± standard error of the mean (SEM) from at least three independent biological replicates (n = 3), with each condition performed in octuplicate. To assess the statistical significance of 1-PPA treatment over time compared to the control, a two-way analysis of variance (ANOVA) followed by Bonferroni’s post hoc test was performed using GraphPad Prism software (v10). A *p*-value <0.05 was considered statistically significant.

Other statistical analyses were performed using GraphPad Prism (GraphPad Software, San Diego, CA, USA). Data were presented as mean ± standard deviation (SD) or ± standard error of the mean (SEM), depending on the experimental context. Comparisons between two groups were performed using an unpaired two-tailed Student’s *t*-test or Welch’s *t*-test (for groups with unequal sample sizes). Multiple group comparisons were performed using one-way analysis of variance (ANOVA) followed by Tukey’s multiple comparisons test. A *p* value < 0.05 was considered statistically significant.

PAR2 expression data were obtained from publicly available glioblastoma patient data and, in particular, they were retrieved from the GlioVis web application (https://gliovis.bioinfo.cnio.es/) [70], including the TCGA [26], CGGA [36], Murat (GSE7696; [27]), Gravendeel (GSE16011; [30]) and Rembrandt (GSE68848; [31]) datasets. PAR2 expression within different glioblastoma tissue layers (GSE113512) was retrieved from a previously published study [28].

Kaplan–Meier survival analysis was achieved by subgrouping patients with available survival data according to their higher or lower (than the median) expression of PAR2. *p* values were calculated by the log rank (Mantel–Cox) test.

## 5. Conclusions

While these preclinical results are promising, several important questions remain to be addressed in future studies, including in vivo efficacy trials to evaluate the effect of 1-PPA in orthotopic glioblastoma xenograft models. In addition, exploring synergistic combinations of 1-PPA with standard-of-care therapies in vivo (e.g., radiation, TMZ) or emerging targeted agents in vivo could lead to more effective treatment regimens and, thus, deserve further investigation.

In conclusion, our study provides preclinical evidence that supports the further development of 1-PPA as a novel therapeutic agent for glioblastoma. By effectively distributing to the brain and targeting the PAR2/SerpinB3 signaling axis, 1-PPA may represent a promising approach to mitigate infiltrative growth and enhance therapy response in glioblastoma. Nevertheless, these findings will need further validation in more complex preclinical disease models before they can be considered to have significant translational potential.

## Figures and Tables

**Table 1:** Non-compartmental PK parameters of 1-PPA following a single i.p. injection of 0.7 mg/kg to BALB/c wild-type male mice (n = at least 4 for each sampling time point).

PK Parameters	Serum	Brain
T_1/2λz_	1.4 (h)	1.6 (h)
T_max_	0.083 (h)	0.5 (h)
C_max_	1751 ± 144 ^##^ (µg/L)	31 ± 3 ^##^ (µg/kg)
AUC _(0–t)_	2345 ± 152 ^##^ (h × µg/L)	131 ± 9 ^##^ (h × µg/kg)
AUC _(0–∞)_	2384 (h × µg/L)	135 (h × µg/kg)
MRT _(0–∞)_	1.8 (h)	3.2 (h)

T_1/2λz_ = elimination half-life; T_max_ = peak time; C_max_ = peak concentration; AUC = area under concentration–time curve; MRT = median residence time; mean ± ^##^ SEM.

**Table 2:** Non-compartmental PK parameters of 1-PPA in serum after a single oral gavage administration of 0.7 mg/kg to BALB/c wild-type male mice (n = 4).

PK Parameters	Serum
T_1/2λz_	1.5 (h)
T_max_	0.5 (h)
C_max_	373 ± 97 (µg/L)
AUC _(0–t) ##_	946 ± 99 (h × µg/L)
AUC _(0–∞)_^##^	965 (h × µg/L)
MRT _(0–∞)_	2.3 (h)
F%	40.5

T_1/2λz_ = elimination half-life; T_max_ = peak time; C_max_ = peak concentration; F = relative bioavailability (AUC _0–∞ h os_/AUC _0–∞ h i.p._). Mean ± ^##^ SEM.

**Table 3:** Tissue-to-serum ratio data obtained following a single i.p. injection of 0.7 mg/kg 1-PPA to BALB/c wild-type male mice.

Tissue	AUC_0–4 h (tissue)_ (h × µg/kg)	Ratio^#^ (t = 4 h)
Brain	90	0.044
Heart	2260	0.065
Kidney	6099	3.010
Liver	1083	0.503
Lung	1876	0.926
Spleen	2445	1.208

Ratio^#^ = AUC_0–4 h (tissue)_/AUC_0–4 h (serum);_ AUC_0–4 h (serum)_= 2024 (h × µg/L).

## Data Availability

Glioblastoma patient data, including the TCGA, CGGA, Murat (GSE7696), Gravendeel (GSE16011), and Rembrandt (GSE68848) datasets, are accessible through the GlioVis web application (https://gliovis.bioinfo.cnio.es/, accessed on 3 April 2026). PAR2 expression within different glioblastoma tissue layers (GSE113512) was retrieved from a previously published study. Additional data are provided as Supplementary Material. Additional data are available upon request.

## Supplementary Materials

The following supporting information can be downloaded at: https://www.mdpi.com/article/10.3390/ijms27167240/s1.

## Abbreviations

1-PPA	1-piperidine propionic acid
AUC	Area under the concentration–time curve
BBB	Blood–brain barrier
C/EBP-β	CCAAT enhancer-binding protein beta
CNS	Central nervous system
FBS	Fetal bovine serum
GSCs	Glioblastoma stem cells
H_2_O_2_	Hydrogen peroxide
HRP	Horseradish peroxidase
i.p.	Intra-peritoneal
MMPs	Matrix metalloproteinases
MRT	Median residence time
NCA	Non-compartmental analysis
PAR2	Protease-activated receptor 2
PK	pharmacokinetic
p.o.	Per os
RWD	Relative Wound Density
SD	Standard deviation
SEM	Standard error of the mean
TME	Tumor microenvironment
TMZ	Temozolomide
UPS	Ubiquitin-proteasome system
