# Supplementary material for: 1-Piperidine Propionic Acid Inhibits PAR2/SerpinB3 Signaling and Reduces Glioblastoma Tumor Aggressiveness
Source: Int J Mol Sci. 2026 Aug 13;27(16):7240. doi: 10.3390/ijms27167240 (PMC13513849; doi:10.3390/ijms27167240)
Supplement: Supplementary file 1 [file ijms-27-07240-s001.zip › ijms-4400065-supplementary.pdf]

SUPPLEMENTARY MATERIAL

Supplementary Materials and Methods

Primary GBM Cell Isolation, Culture, and Maintenance

Primary glioblastoma specimens were obtained from patients undergoing surgical resection at the University Hospital of Padova, following approval by the local Ethics Committee and in accordance with the Declaration of Helsinki. Written informed consent was obtained from all patients prior to tissue collection.

Tumor samples were enzymatically and mechanically dissociated to generate single-cell suspensions. Cells were seeded onto fibronectin-coated culture plates and maintained as adherent monolayers in DMEM/F12 medium (Biowest, Nuaillé, France) supplemented with 10% BIT9500 serum substitute (STEMCELL Technologies, Vancouver, Canada), 20 ng/mL basic fibroblast growth factor (bFGF), and 20 ng/mL epidermal growth factor (EGF) (both from Cell Guidance Systems Ltd., Cambridge, UK).

Cells were plated at a density of 1×10^4 cells per well and cultured under hypoxic conditions (2% O_2, 5% CO_2, balanced N_2) using an H35 hypoxic workstation (Don Whitley Scientific Ltd., Shipley, UK) to better recapitulate the hypoxic tumor microenvironment characteristic of GBM.

Both primary cultures were characterized by wild type *IDH* genes and unmethylated *MGMT* gene promoter.

Live-cell imaging experiments, performed using the IncuCyte® S3 system, were conducted under standard incubation conditions (5% CO_2, atmospheric O_2). These conditions were maintained consistently across all IncuCyte-based assays to ensure methodological comparability among experiments.

Live-Cell Imaging Assays in primary Glioblastoma cells

Live-cell imaging analyses were performed on primary cells using the IncuCyte S3 Live-Cell Analysis System (Sartorius AG, Göttingen, Germany).

Cell Proliferation in primary Glioblastoma cells

Cells were seeded at a density of 8×10^3 cells per well in 96-well plates and allowed to adhere for 18–24 h before treatment with increasing concentrations of 1-PPA. Each experimental condition was performed in octuplicate wells.

Phase-contrast images were acquired every 2 h for 5 days using a 10x objective with the IncuCyte® S3 Live-Cell Analysis System. Cell proliferation was quantified using IncuCyte software (2024A) by measuring cell confluence over time and reported as fold change relative to baseline (t0).
Sulforhodamine B (SRB) staining was performed by seeding 25 x 10^3 cells per well in 24-well plates and allowed to adhere for 18–24 h before treatment with 100 ng/µl of 1-PPA, 400 µM TMZ, or the combination of them. Following 72 h of treatment, cells were fixed with ice-cold 10% (w/v) trichloroacetic acid and incubated overnight at 4°C. Cells were subsequently washed with distilled water, air-dried at room temperature, and stained with 0.4% (w/v) Sulforhodamine B (SRB) (MedChemExpress) solution for 15 min. Excess dye was removed by repeated washes with 1% (v/v) acetic acid. Following solubilization of the protein-bound SRB dye in 10 mM Tris base solution (Carlo Erba), the absorbance was measured at 510 nm using a Spark multimode microplate reader (Tecan).

Pharmacokinetics and organ distribution of 1-PPA *in vivo*

Blood samples were collected 0.083, 0.025, 0.500, 1, 2, 3, 4, 6 and 9 hours after 1-PPA administration by tail bleeding into sterile tubes Eppendorf®, kept refrigerated at +4ºC and centrifuged at 1520 g for 10 minutes at room temperature. Collected serum samples were frozen at -80ºC until use.
To evaluate the organ distribution of 1-PPA, the compound was injected intraperitoneally (0.7 mg/Kg) in mice and the tissues of interest (brain, kidney, spleen, lung, liver and heart) were collected at 0.083, 0.250, 0.500, 1 and 4 hours post-injection, snap-frozen in liquid nitrogen, and stored at -80 ºC until use. Brain tissue was additionally harvested at 6 and 9 hours to further assess the biodistribution of 1-PPA in the central nervous system.
Mice were bred at the Animal Care Facility of the Experimental Surgery Division of the University of Padua.
For mass spectrometry analysis, the following steps were carried out:

1. <u>Sample preparation and extraction.</u> Both liquid-liquid extraction and solid-phase extraction (SPE) were tested for quantitative recovery of PPA from the matrices. Different organic solvents, including methanol, acetonitrile, ethyl acetate, and dichloromethane, as well as various types of SPE cartridges (Oasis WAX, MCX, and WCX; 1 cc/30 mg; Waters Corp., Milford, MA, USA) were tested for optimal recovery. The procedure which gave the best results in terms of mean recovery (i.e., 95.7% for serum and 98.8% for brain homogenate) is described below and involves the use of the Oasis PRiME MCX SPE columns (1cc/30 mg; Waters Corp.).

The columns were conditioned with 1 mL of MeOH and 1 mL of water before use. Fifty microliters of 4% H3PO4 were added to 50 μL of mouse serum and the mixture was stirred well before being added into an activated SPE column. The loaded cartridges were washed with 1 mL of 100 mM Ammonium formate containing 2% formic acid, followed by 1 mL of MeOH. Afterward, they were eluted with a 5% ammonium hydroxide solution in MeOH (1 mL). The obtained eluates were dried under an air stream at 50°C using a TurboVap evaporator (Zymarck, Hopkinton, MA, USA) and the resulting residues were reconstituted in 100 μL of water/0.1% formic acid in MeOH (50:50, v/v), vortexed and centrifuged for 10 min at 13,684g. The resulting supernatants were then transferred to the vials and 10 μL were injected into the LC-MS/MS system.

Whole-brain, kidney, liver, lung, spleen and heart samples were thawed and weighed before being manually grounded with 1 mL of water using a small pestle and a mortar. The tissue homogenates were then vigorously vortexed and centrifuged at 13,684*g* for 20 min at 4°C. The supernatant was then pooled and stored at −80°C until use. An aliquot of 50 μL was diluted with 50 μL of 4% H_3PO_4 and processed as described for serum samples (see above).

2. <u>Standard stock solution, calibrators and quality control sample preparation.</u> 1-PPA was dissolved in methanol to obtain a stock solution at 1 mg/mL. Working solutions of 1-PPA used to spike blank samples were prepared by appropriate dilutions of the concentrated stock standard solution with mobile phase (0.1% formic in H_2O:0.1% formic acid in methanol, 80:20).

   Calibrators and quality control samples (QC) were obtained by spiking a pool of blank mouse serum or blank mouse brain homogenate obtained from untreated animals with 5 μL of working solutions of 1-PPA. Six different calibration curves, ranging from 1 to 500 ng/mL and including the LLOQ, were constructed on six different days to evaluate the linearity of the method. QC samples were used to evaluate the extraction efficiency of PPA (recovery) from serum and tissue homogenates by comparing the peak areas of 1-PPA added into blank matrices before the extraction procedure with those obtained for 1-PPA added, at the same concentrations, to the blank matrices after the extractions. The limit of quantification (LOQ) of 1-PPA was defined as the lowest calibrator concentration on the curve that could be quantified with a precision within 20%, and the trueness between 80% and 120%. In the concentration range considered (1-500 ng/mL), a linear relationship between the analyte's peak area and the corresponding concentration was achieved for both serum and brain (R^2 ≥0.99). The LOQ was fixed at 1 ng/mL for all matrices.

3. LC-MS/MS analysis. The chromatographic separation was achieved using an Accela 600 HPLC pump with a CTC automatic injector (Thermo Fisher Scientific, San Jose, CA, USA) equipped with a Pursuit 5 C18 (50×4.6 mm, 5 µm; Agilent, CA, USA) analytical column. A gradient profile was applied using water with 0.1% formic acid (v/v) as solvent A and MeOH with 0.1 % formic acid (v/v) as solvent B. The mobile phase composition (%A: %B) was as follows: 75:25 from 0 to 3 min, 10:90 at 5 min, held at 10:90 until 6 min, and then 75:25 from 7 to 9 min to re-equilibrate the system. The injection volume was 10 µL, and the flow rate was set at 300 µL/min. Under the above chromatographic conditions, the retention time of 1-PPA was 2.04 min. The mass detection was achieved by an LTQ XL ion trap (Thermo Fisher Scientific, San Jose, CA, USA) equipped with a heated electrospray ionization (HESI-II) probe. The ESI source was used in the positive ion mode. The mass analyzer was set in the full scan monitoring mode. The 1-PPA standard solution at 1 µg/mL was infused directly into the mass spectrometer via a syringe pump at a 20 µL/min flow rate to find the fragmentation patterns and tuning parameters. The mass spectrometer was operated using ESI by monitoring the transition pair (precursor to product ion) of m/z 159-98 for 1- PPA (collision energy: 35 %) in the positive ion mode. The optimum tuning parameters used were as follows: sheath gas flow, 50 arbitrary units; auxiliary gas flow, 4 arbitrary units; ion spray voltage, 3 kV; capillary temperature, 275°C; capillary voltage, 5 V; and tube lens, 40 V. The Xcalibur software (version 2.1) was used for system control, data acquisition, and analysis.

4. Assessment of blood-brain barrier (BBB) permeability. BBB permeability of 1-PPA was estimated by calculation of both *in silico* predicted logarithm of blood–brain ratio (LogBB) and experimental (*in vivo*) LogBB; the latter approach has been widely used in the pharmaceutical industry to support drug discovery programs (doi: 10.1093/bioinformatics/btad577).

5. In silico Predicted LogBB. *In silico* predicted LogBB value of 1-PPA and temozolomide was obtained using the free software available at http://ssbio.cau.ac.kr/software/logbb_pred/. This quantitative model is based on machine learning and used the Light Gradient Boosting Machine (LightGBM) algorithm to develop a regression model to predict BBB permeability; the model was trained using a dataset of 1,276 experimentally measured LogBB values retrieved from the open literature (doi: 10.1093/bioinformatics/btad577).

6. In vivo LogBB. *In vivo* LogBB value of 1-PPA was calculated according to the following equation:

$$LogBB = Log\left(\frac{C_{Brain}}{C_{Blood}}\right)$$

where C_{Brain} and C_{Blood} are the concentration of the test compound in the brain and blood serum, respectively, determined by LC-MS/MS analysis.

Supplementary Figures

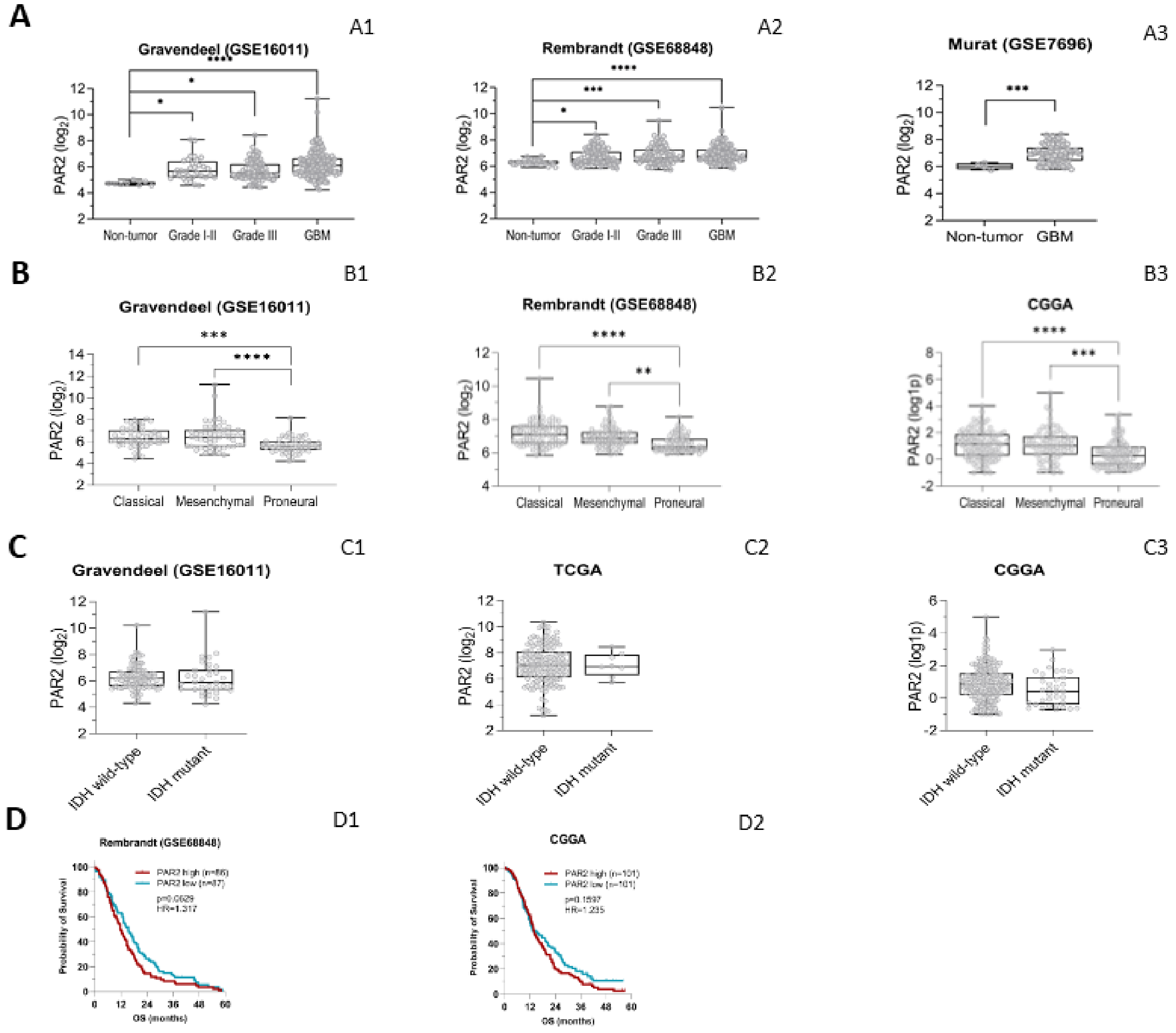

Supplementary Figure S1. (A) Boxplots displaying the mRNA expression of PAR2 in gliomas and non-tumor specimens from the Gravendeel (GSE16011; n=284; (A1)), Rembrandt (GSE688848; n=343; (A2)) and Murat (GSE7696; n=84; (A3) datasets. (B) Boxplots displaying the mRNA expression of PAR2 according to different Glioblastoma molecular subtypes from the Gravendeel (GSE16011; n=159; (B1)), Rembrandt (GSE688848; n=219; (B2)) and CGGA (n=287; (B3) datasets. (C) Boxplots displaying the mRNA expression of PAR2 according to the *IDH* gene status in patients from the Gravendeel (GSE16011; n=128; (C1)), TCGA (n=150; (C2)) and CGGA (n=216; (C3)) datasets. (D) Kaplan-Meyer curves showing the impact of the expression of PAR2 (higher versus lower than the cohort median) in Glioblastoma patients from the Rembrandt (GSE688848; (D1) or the CGGA (D2) datasets. *p<0.05, **p<0.01; ***p<0.001, ****p<0.0001 by one-way ANOVA with Tukey's multiple comparisons test or t-test with Welch's correction. Survival analysis by Logrank (Mantel-Cox) test.

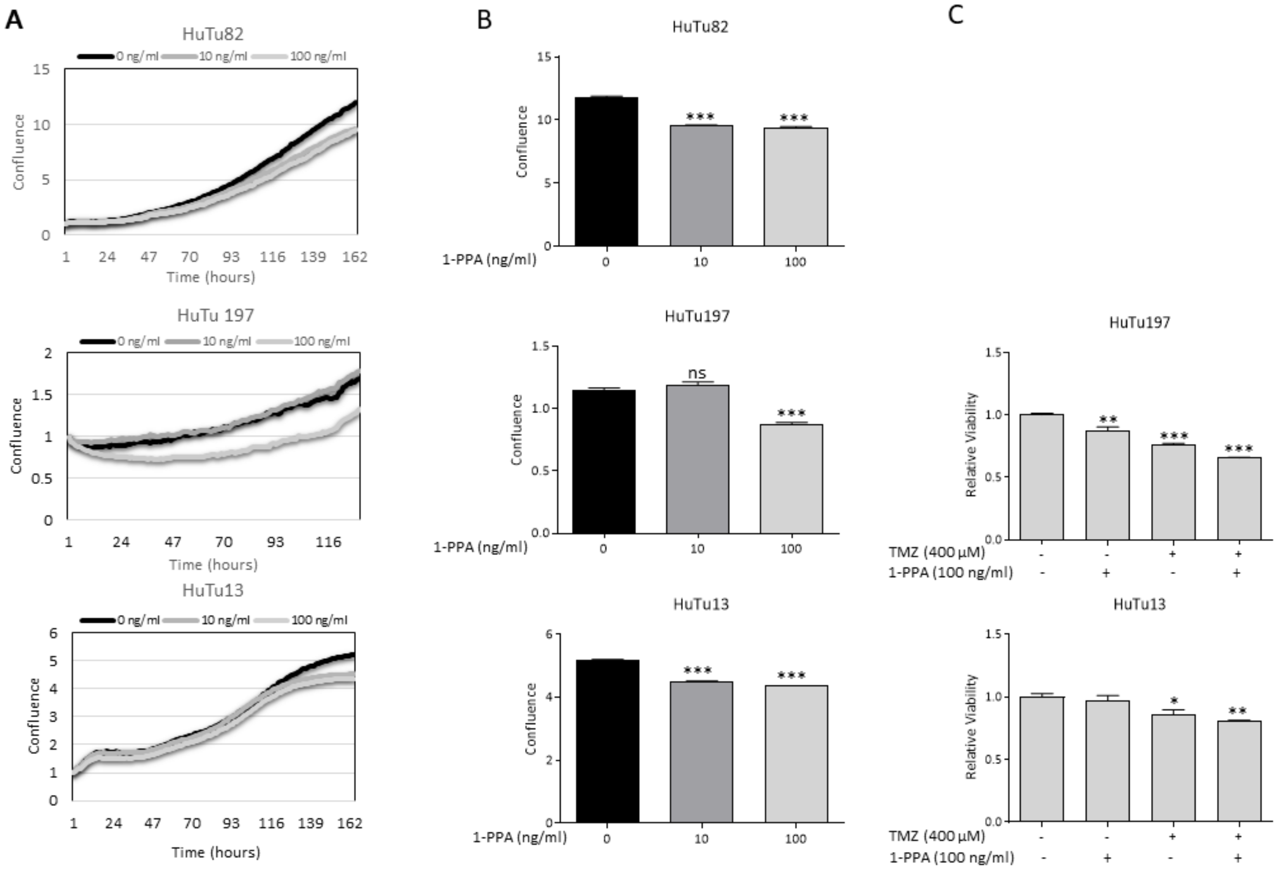

Supplementary Figure S2. Effect of 1-PPA on the proliferation of primary glioblastoma cells. Cell proliferation was monitored in three different Glioblastoma primary cultures (HuTu82, HuTu197, and HuTu13) using the IncuCyte® S3 Live-Cell Analysis System and expressed as fold change in confluence relative to t0 over a 5-day treatment period in presence of increasing concentrations of 1-PPA. Panel (A) shows real-time cell proliferation in the different cell lines. Panel (B) shows histograms at day 5 are represented as the mean ± SD of three independent experiments, each performed in octuplicate wells. Panel (C) shows comparison of relative cell number (SRB staining) of cells treated for 72 h either with 1-PPA, TMZ or the combination of them at the indicated concentrations. Statistical analysis was performed using a two-way ANOVA followed by Bonferroni's multiple comparisons test or one-way ANOVA with Tukey’s multiple comparison test. ns, not significant; *$p<0.05$; **$p<0.01$; ***$p<0.001$.

Supplementary Tables

Table S1: Mean (± SD; ng/mL) of 1-PPA concentration data for each sampling time point (h) with CI of the mean.

Sampling time (h)	Mean (n = at least 4, ng/mL)	SD	Lower 95% CI of mean	Upper 95% CI of mean
0.083	1751	381.3	1398	2104
0.25	1252	553	933	1572
0.5	1019	336	793	1244
1	804	437	551	1057
2	352	86	272	432.5
3	112	38	77	147
4	190	50	143	230
6	41	39	19	63
9	19	19	1	37

Table S2: List of primary antibodies used for Western blot analysis.

Target Antigen	Host Species	Clonality	Catalog number	Manufacturer
SerpinB3	Mouse	Monoclonal	CF506877	Origene
PAR2	Rabbit	Monoclonal	ab180953	Abcam
ERK1/2	Mouse	Monoclonal	sc-514302	SantaCruz
Phospho ERK1/2	Rabbit	Polyclonal	9101	Cell Signaling
α-Tubulin	Rabbit	Polyclonal	11224-1-AP	Proteintech

Table S3: List and sequences of human primers used for quantitative real-time PCR.

Gene	Sequence
Hu_45s	For: 5’-CGGCTACCACATCCAAGGAA-3’<br>Rev: 5’-GCTGGAATTACCGCGGCT-3’
Hu_SB3	For: 5’-GCAAATGCTCCAGAAGAAAG-3’<br>Rev: 5’-CGAGGCAAAATGAAAAGATG-3’
Hu_PAR2	For: 5’-GCTAGCAGCCTCTCTCTCCT-3’<br>Rev: 5’-GTGGGATGTGCCATCAACCT-3’
